# Preoperative vitamin D level does not affect the short-term functional outcome after total knee arthroplasty in elderly women

**DOI:** 10.1186/s43019-020-00050-7

**Published:** 2020-06-11

**Authors:** Il Yeong Hwang, Ki Bong Park, Sung Who Chang, Sung Do Cho, Yoon Seok Youm

**Affiliations:** 1 Department of Orthopedic Surgery, Ulsan University Hospital, University of Ulsan College of Medicine, Ulsan, South Korea; 2 Department of Orthopedic Surgery, Dongcheondongkang Hospital, Ulsan, South Korea; 3 Department of Orthopedic Surgery, Seongnam Citizens Medical Center, 10, Sujeong-ro 171beon-gil, Sujeong-gu, Seongnam-si, Gyeonggi-do 13290 South Korea

**Keywords:** Vitamin D, Arthroplasty, Knee, Outcome assessment, Women

## Abstract

**Background:**

We aimed to evaluate the effect of vitamin D levels on the functional outcome of elderly women who underwent total knee arthroplasty (TKA).

**Methods:**

Seven hundred and four patients (1013 knees) who underwent primary TKA were included in our retrospective study. Preoperative vitamin D levels were measured and the relationship analyzed between these and age, weight, height, body mass index, and bone mineral density. Two hundred and twenty patients (220 knees) who received unilateral TKA and were followed up for more than 1 year after operation were divided into two groups: Group 1, serum 25-hydroxyvitamin D3 (25(OH)D3) level < 20 ng/ml; and Group 2, 25(OH)D3 level ≥ 20 ng/ml. Both groups were evaluated for the relationship between vitamin D levels and postoperative Visual Analogue Scale (VAS) score, Knee Society Knee Score (KSKS), Knee Society Function Score (KSFS), and Western Ontario and McMaster Universities Arthritis Index (WOMAC) score.

**Results:**

The number of vitamin D-deficient patients (< 20 ng/ml of serum 25(OH)D3 level) was 556 (79.0%). In the correlation analysis, the vitamin D level was negatively correlated with weight only (*p* = 0.033). No significant differences were observed between the groups in terms of postoperative VAS score, KSKS, KSFS, and WOMAC score.

**Conclusions:**

Vitamin D deficiency was highly prevalent in patients who underwent TKA. Vitamin D levels negatively correlated with weight. Low vitamin D level was not a risk factor for unsatisfactory TKA outcome in elderly women.

## Background

Total knee arthroplasty (TKA) is one of the most successful interventional procedures to improve the health of patients [1]. Based on successful results, the popularity of TKA is increasing worldwide [2]. Nonetheless, some procedures have shown unsatisfactory results and many studies have been conducted to identify and resolve any shortcomings. Various factors such as age, sex, diagnosis, body mass index (BMI), and comorbidities have been reported to affect the outcome of TKA [3–8].

Vitamin D is well known to play an important role in the bone and mineral metabolism of the body, and a deficiency of Vitamin D can cause rickets in children, osteopenia in adults, osteoporosis, and fractures. Vitamin D deficiency has been noted to be relatively common worldwide; according to the various criteria of vitamin D 25-hydroxyvitamin D3 (25(OH)D3) deficiency, the prevalence rate is approximately 47–93% in Korea [9–12].

Osteoarthritis is a disease that causes the inflammation and deformation of joint cartilage and the synovial and peripheral synovial membrane [13]. Vitamin D is also implicated in the articular cartilage, bone, muscle, and various inflammatory responses, and the claim that a lack of vitamin D may affect the pathogenesis of osteoarthritis has been raised [11, 14, 15].

Low vitamin D levels have been reported to adversely affect functional outcomes after hip arthroplasty, and it has been reported that vitamin D deficiency in patients with knee arthroplasty is associated with a high complication rate, poor functional state before and after surgery, and long hospital stay [16–22]. However, the effect of vitamin D level on function after arthroplasty is still controversial.

However, the Korean female population has the lowest absolute concentration of serum 25(OH)D3 compared with other countries, and the prevalence of vitamin D deficiency is higher in elderly women. The criterion for vitamin D deficiency is also different (25(OH)D3 level < 20 ng/ml) [23–25]. For these reasons, we thought that it is necessary to study whether the vitamin D level affects the functional outcome after total knee arthroplasty in Korean elderly women.

We hypothesized that the vitamin D level would affect the short-term functional outcome after total knee arthroplasty in Korean elderly women.

## Methods

This study was approved by the institutional review board of our hospital.

### Subjects

A total of 704 patients (1013 knees) who underwent primary total knee arthroplasty from February 2012 to December 2015 by one surgeon were included in our study. Preoperative vitamin D levels were measured the day before operation, regardless of whether or not the patient had vitamin D intake. The mean age of the 704 patients (1013 knees) was 69.4 ± 7.0 years (range 41–87 years). Seventy-six patients (10.8%) were male and 628 patients (89.2%) were female. The number of vitamin D-deficient patients (< 20 ng/ml of serum 25(OH)D3) was 556 (79.0%). The prevalence of vitamin D deficiency and the correlation between vitamin D and baseline characteristics were investigated.

Then, to further investigate the relationship between functional scores and vitamin D levels, we excluded patients who could affect the functional score. First, 76 male patients (94 knees) were excluded. Afterward, another 291 patients (582 knees) who underwent bilateral surgery during the study period were excluded because one side of the operation could influence the pain and rehabilitation of the other side. Also, patients with postoperative complications that might affect the rehabilitation process (four patients and four knees) and patients that underwent reoperation (one patient and one knee) were excluded. Another 112 patients (112 knees) who were not followed up for 1 year after the surgery were excluded. Finally, 220 female patients (220 knees) who received unilateral TKA and were followed up for more than 1 year after the operation were included in the analysis of the relationship between vitamin D levels and functional score. Vitamin D deficiency was defined as a serum 25(OH)D3 level of less than 20 ng/ml and participants were divided into two groups: group 1, 25(OH)D_3_ level < 20 ng/ml; and group 2, ≥ 20 ng/ml) [23]. Among the 220 female patients included in the comparative study, 44 patients without vitamin D deficiency belonged to group 1 and 176 patients with vitamin D deficiency belonged to group 2. Vitamin D showed significant differences between group 1 and group 2, but there was no significant difference in age, weight, height, BMI, BMD, and side (Table 1).

**Table 1:** Comparison of demographics between the two groups

Variable	Group 1 (*n* = 44)	Group 2 (*n* = 176)	*p* value
Vitamin D level (ng/ml)^*^	27.8 (20.2–52.6)	11.4 (2.0–19.9)	0.001
Age (years)	69.6 (55.0–84.0)	67.4 (46.0–86.0)	0.055
Weight (kg)	60.2 (39.0–86.4)	62.7 (42.3–98.0)	0.101
Height (cm)	151 (142–164)	153 (139–177)	0.143
BMI (kg/m^2^)	26.2 (19.3–34.2)	26.8 (18.0–37.5)	0.279
BMD	−2.3 (− 3.1 to –1.1)	−2.3 (− 3.3 to –0.7)	0.601
Right side	24 (54.5%)	76 (43.2%)	0.176

Data for age, weight, height, BMI, and BMD displayed as mean (range). Data for right side displayed as number (percentage)

*BMD* bone mineral density, *BMI* body mass index

^*^Statistically significant

### Investigated variable

The preoperative serum 25(OH)D3 level was measured, and their medical records were reviewed for age, sex, height, weight, body mass index (BMI), bone mineral density (BMD), Visual Analogue Scale (VAS) score, Knee Society Knee Score (KSKS), Knee Society Functional Score (KSFS), and Western Ontario and McMaster Universities Arthritis Index (WOMAC) score. The BMD was assessed using dual-energy X-ray absorptiometry (Delphi-W; Hologic, Waltham, MA, USA), which included the first to fourth lumbar spine segments and the proximal femur. The representative value of BMD was taken from the lowest *T*-score of the proximal femur and the average of the two lowest scores of the lumbar spine. The VAS score, KSKS, KSFS, and WOMAC score were measured again 1 year after surgery.

### Surgical and rehabilitation method

A single senior surgeon performed all surgeries. Skin incisions were at the midline, and midvastus arthrotomy was done for all patients. The posterior cruciate was sacrificed, and Advance Medial Pivot Total Knee Arthroplasty (Wright Medical Technology, Arlington, TN, USA) and NexGen Legacy posterior-stabilized (LPS)-flex fixed TKR system (Zimmer, Warsaw, IN, USA) implants were used. Cement was used for implantation in all cases. Immobilization was performed using an extension brace on the first postoperative day and patients were allowed to undertake quadriceps strengthening exercises and active straight leg-raising as instructed prior to surgery. On the second postoperative day, continuous passive motion exercises were initiated. At 3–4 days after surgery, when straight leg-raising could be performed without difficulty, progressive weight bearing was started with the assistance of a walker or a cane.

### Statistical analysis

Data were presented as means with ranges. All of the continuous variables (preoperative serum 25(OH)D3, age, height, weight, BMI, BMD, VAS score, KSKS, KSFS, and WOMAC score) are accepted to be normality assumptions based on the Kolmogorov–Smirnov test and parametric tests were performed. Pearson correlation was used to analyze the correlation between vitamin D level and various variables. The two-sample *t* test and chi-square test were performed for the comparison between the groups. In addition, an analysis of covariance (ANCOVA) was performed to control the variables with a significant correlation with the vitamin D level. *p* < 0.05 was regarded as statistically significant. Statistical analysis was performed using SPSS version 21 (SPSS Inc., Chicago, IL, USA).

## Results

### Correlation between vitamin D level and other variables

Before comparing the postoperative results of the two groups, correlation analysis was performed to identify the variables that could disturb the level of vitamin D. There was no correlation between preoperative vitamin D levels and age, height, BMI, and BMD. However, preoperative vitamin D levels showed a negative correlation with weight (Table 2).

**Table 2:** Results of Pearson correlation between vitamin D level and age, height, weight, BMI, and BMD

Variable	Age (years)	Weight (kg)*	Height (cm)	BMI (kg/m^2^)	BMD
Vitamin D level (ng/ml)	69.4 (41–87)	62.5 (32.5–99.0)	153 (133–178)	26.7 (16.0–38.9)	−2.3 (− 3.3 to –0.7)
*r*	0.005	−0.096	−0.011	− 0.011	0.004
*p* value	0.870	0.002	0.735	0.718	0.894

Data for vitamin D level displayed as mean (range)

*BMD* bone mineral density, *BMI* body mass index, r correlation coefficient

*Statistically significant

### Preoperative and postoperative results of the two groups with and without vitamin D deficiency

The preoperative VAS score, KSKS, KSFS, and WOMAC score showed no significant differences between the two groups and significantly improved after operation in both groups. Regarding the correlation between weight and vitamin D levels, the postoperative VAS score, KSKS, KSFS, and WOMAC score indicated no significant variations between the two groups (Table 3).

**Table 3:** Comparison of functional outcome between vitamin D-sufficient group (group 1) and vitamin D-deficient group (group 2)

Variable	Group 1 (*n* = 44)	Group 2 (*n* = 176)	*p* value* w/o control	*p* value† w control
Preoperatively				
VAS score	61.3 (50–80)	60.8 (50–80)	0.734	0.612
KSKS	41.6 (15–62)	39.7 (12–58)	0.185	0.150
KSFS	70.8 (40–85)	70.4 (32–82)	0.863	0.695
WOMAC score	44.2 (36–68)	42.3 (32–67)	0.768	0.691
Postoperative 1 year				
VAS score	44.5 (20–50)	44.5 (10–50)	0.983	0.885
KSKS	92.1 (55–100)	92.2 (45–100)	0.948	0.941
KSFS	85.8 (50–100)	88.4 (45–100)	0.338	0.394
WOMAC score	8.2 (0–32)	10.3 (0–47)	0.287	0.286

Data for VAS score, KSKS, KSFS, and WOMAC score displayed as mean (range)

*KSFS* Knee Society Functional Score, *KSKS* Knee Society Knee Score, *VAS* Visual Analogue Scale, *WOMAC* Western Ontario and McMaster Universities Arthritis Index

*Comparison with two-sample *t* test without control of weight

†Comparison with analysis of covariance, as weights were assigned to covariate variable

## Discussion

The most important finding of the present study is that there is no relationship between preoperative vitamin D levels and short-term functional score after TKA.

Until now, several functions of vitamin D have been identified; therefore, much research has been conducted to check whether vitamin D affects arthroplasty [11, 17–19, 21, 22, 26, 27]. Jansen and Haddad [17] showed that vitamin D-deficient patients with a serum 25(OH)D3 level of less than 40 nmol/l represented 24% of 139 patients who underwent TKA and that the preoperative KSKS was significantly lower in patients with vitamin D deficiency. However, elderly women in Korea have a higher prevalence of vitamin D deficiency than those in other regions where results have been reported [23, 25]. In the case of Korean older adults, the criterion for vitamin D deficiency is different (25(OH)D3 level < 20 ng/ml) [23]. Vitamin D deficiency was also found to be associated with significantly low WOMAC scores and longer hospital stays [18]. Kelly et al. [26] conducted a study of Irish patients using a criterion (< 50 nmol/l of serum 25(OH)D3 level) similar to our study (< 20 ng/ml of serum 25(OH)D3 level), and found that 40% of patients who underwent TKA had vitamin D deficiency. In the present study, vitamin D-deficient patients with a serum 25(OH)D3 level of ≤ 20 ng/ml represented 78.1%, showing a higher prevalence rate than in other regions. Similar findings were reported in some Korean studies [10, 25]. In this study, the functional VAS score, KSKS, KSFS, and WOMAC score were not related to vitamin D deficiency. Further studies are needed to establish the role of vitamin D in regions with a high prevalence of vitamin D deficiency such as Korea.

Vitamin D deficiency affecting the functional score after TKA has been reported in previous studies [17, 18, 20]. In previously published studies, the functional scores were measured at 3 months or 6 months after surgery [17, 19, 20]. This study showed that vitamin D deficiency did not affect the functional score at 1 year postoperatively. Further research is needed to determine whether the timing of the measurement influences the outcome as there is a difference in the measurement period between the previous study and this study.

There is still some debate as to what level of vitamin D is regarded as sufficient. There are various reports on the criteria for dividing vitamin D insufficiency and deficiency. Hwang et al. [23] have suggested that a serum 25(OH)D3 level of ≥ 20 ng/ml (= 50 nmol/l) is sufficient for adults over 49 years of age in Korea. Therefore, in this study, vitamin D deficiency was defined as a serum 25(OH)D3 level of less than 20 ng/ml.

It has been reported that vitamin D-deficient patients showed similar functional score to the vitamin D-sufficient patient group after a total joint replacement with vitamin D supplementation [19, 22]. Kelly et al. [26] reported that vitamin D levels significantly decreased after TKA and this could be overcome by vitamin D supplementation. Further research is needed to confirm whether vitamin D supplementation can produce a better functional outcome.

There are several limitations of this study. First, there is a large difference in the sample size between the two groups because the prevalence of vitamin D deficiency is higher in the Korean population than in other populations. Further prospective studies in age-matched populations are necessary. Second, vitamin D is also highly prevalent related to parathyroid hormone, binding protein level, and seasonal effect, and is also related to the timing and amount of blood sampling before surgery, so studies to control these variables are needed.

## Conclusions

Vitamin D deficiency was highly prevalent in patients who underwent TKA. Vitamin D levels negatively correlated with weight. Low vitamin D level was not a risk factor for unsatisfactory TKA outcome in elderly women.

## Data Availability

Not applicable.

## Abbreviations

TKA: Total knee arthroplasty
BMI: Body mass index
BMD: Bone mineral density
VAS: Visual Analogue Scale
KSKS: Knee Society Knee Score
KSFS: Knee Society Functional Score
WOMAC: Western Ontario and McMaster Universities Arthritis Index
